# Circulatory Serum Krebs von Den Lungen-6 and Surfactant Protein-D Concentrations Predict Interstitial Lung Disease Progression and Mortality

**DOI:** 10.3390/cells12091281

**Published:** 2023-04-28

**Authors:** Meghna Rai, Ashwaghosha Parthasarathi, Narasimha M. Beeraka, Mohammed Kaleem Ullah, Sowmya Malamardi, Sunag Padukudru, Jayaraj Biligere Siddaiah, Chinnappa A. Uthaiah, Prashant Vishwanath, Sindaghatta Krishnarao Chaya, Subramanian Ramaswamy, Swapna Upadhyay, Koustav Ganguly, Padukudru Anand Mahesh

**Affiliations:** 1 Department of Respiratory Medicine, JSS Medical College, JSS Academy of Higher Education and Research, Mysuru 570015, India; 2 Allergy, Asthma, and Chest Centre, Krishnamurthypuram, Mysuru 570004, India; 3 Rutgers Centre for Pharmacoepidemiology and Treatment Science, New Brunswick, NJ 08901-1293, USA; 4 Department of Pharmaceutical Chemistry, JSS College of Pharmacy, JSS Academy of Higher Education and Research, Mysuru 570015, India; 5 Raghavendra Institute of Pharmaceutical Education and Research (RIPER), Chiyyedu, Anantapuramu 515721, Andhra Pradesh, India; 6 Centre for Excellence in Molecular Biology and Regenerative Medicine (A DST-FIST Supported Center), Department of Biochemistry (A DST-FIST Supported Department), JSS Medical College, JSS Academy of Higher Education and Research, Mysore 570015, India; 7 Division of Infectious Disease and Vaccinology, School of Public Health, University of California, Berkeley, CA 94720, USA; 8 School of Psychology & Public Health, College of Science Health and Engineering, La Trobe University, Melbourne 3086, Australia; 9 Yenepoya Medical College, Yenepoya University, Mangalore 575018, Karnataka, India; 10 Department of Clinical Immunology & Rheumatology, JSS Medical College, JSS Academy of Higher Education and Research, Mysuru 570015, India; 11 Unit of Integrative Toxicology, Institute of Environmental Medicine (IMM), Karolinska Institutet, 17177 Stockholm, Sweden

**Keywords:** interstitial lung disease, Krebs von den Lungen-6, surfactant protein D, mortality, progression, KL6, ILD, SP-D

## Abstract

There is a need for biomarkers to predict outcomes, including mortality, in interstitial lung disease (ILD). Krebs von den Lungen-6 (KL-6) and surfactant protein D (SP-D) are associated with lung damage and fibrosis in all ILDs and are related to important clinical outcomes. Though these two biomarkers have been associated with ILD outcomes, there are no studies that have evaluated their predictive potential in combination. This study aims to determine whether KL-6 and SP-D are linked to poor disease outcomes and mortality. Additionally, we plan to examine whether changes in KL-6 and SP-D concentrations correspond with changes in lung function and whether serial measurements improve their predictive potential to identify disease progression and mortality. Forty-four patients with ILD participated in a prospective 6-month longitudinal observational study. ILD patients who succumbed had the highest KL-6 levels (3990.4 U/mL (3490.0–4467.6)) and highest SP-D levels (256.1 ng/mL (217.9–260.0)), followed by those who deteriorated: KL-6 levels 1357.0 U/mL (822.6–1543.4) and SP-D levels 191.2 ng/mL (152.8–210.5). The generalized linear model (GLM) analysis demonstrated that changes in forced vital capacity (FVC), diffusing capacity of lungs for carbon monoxide (DLCO), forced expiratory volume in 1 s (FEV1), and partial pressure of arterial oxygen (PaO_2_) were correlated to changes in KL6 (*p* = 0.016, 0.014, 0.027, 0.047) and SP-D (*p* = 0.008, 0.012, 0.046, 0.020), respectively. KL-6 (odds ratio (OR): 2.87 (1.06–7.79)) and SPD (OR: 1.76 (1.05–2.97)) were independent predictors of disease progression, and KL-6 (hazard ratio (HR): 3.70 (1.46–9.41)) and SPD (HR: 2.58 (1.01–6.59)) were independent predictors of death by Cox regression analysis. Combined biomarkers (KL6 + SPD + CT + FVC) had the strongest ability to predict disease progression (AUC: 0.797) and death (AUC: 0.961), on ROC analysis. Elevated KL-6 and SPD levels are vital biomarkers for predicting the severity, progression, and outcomes of ILD. High baseline levels or an increase in levels over a six-month follow-up despite treatment indicate a poor prognosis. Combining KL6 and SPD with conventional measures yields a more potent prognostic indicator. Clinical studies are needed to test additional interventions, and future research will determine if this combined biomarker benefits different ethnicities globally.

## 1. Introduction

Interstitial lung disease (ILD) is a varied group of disorders leading to interstitial inflammation and fibrosis with significant morbidity and mortality. According to the Global Burden of Disease Study, ILD was ranked 41st in 1990, 32nd in 2010, and 30th in 2019 [1]. ILD’s clinical course is exceedingly diverse and unpredictable. As an illustration, some patients can be stable or have a slow decline in pulmonary function. Others can exhibit sudden worsening of ILD, faster deterioration, or a substantial increase in defects on high-resolution computed tomography (HRCT) within a short period [2,3].

Pulmonary function tests (PFTs), chest X-rays, high-resolution chest computed tomography (HRCT), and, if necessary, lung biopsies are the primary tools used to diagnose and prognosticate ILD [3,4]. While these tools may be advantageous in diagnosing ILD, they fail as serial prognostic indicators as these measurements are not always sensitive due to observer bias and the need for patient cooperation. Additionally, frequent scans expose patients to increased radiation, and collecting recurrent lung samples becomes unfeasible due to its invasive nature. Furthermore, environmental and cultural variables such as paucity of resources complicate ILD diagnosis in low- to middle-income countries [5]. Biomarkers have the potential to respond quickly to minor changes, are both sensitive and specific to a disease, are widely accessible, and are relatively inexpensive [6]. It has been extensively used for various respiratory conditions such as community-acquired pneumonia, acute lung injury and respiratory distress syndrome, interstitial lung disease, chronic obstructive pulmonary disease, COVID-19, and asthma [7,8,9,10].

Krebs von den Lungen-6 (KL-6) and surfactant protein D (SP-D) are part of the innate immune system. KL6, produced by type II alveolar epithelial cells and bronchial epithelial cells and first described by Kohno et al. in 1985, is a glycoprotein produced by the MUC1 gene [11,12]. KL-6 is a marker of epithelial damage and has been studied as a biomarker for ILD diagnosis and prognosis, including a decline in lung functions and mortality, as well as response to treatment [13,14,15,16]. Clara cells and alveolar epithelial cells both generate SP-D, a collection of the CC-type lectin superfamily [17]. In a normal lung, they are mostly dispersed on the surface of type II alveolar epithelial cells and respiratory bronchiolar epithelial cells. When they are translocated on extrapulmonary epithelial surfaces or in serum, they can be utilized as biomarkers for pulmonary disease states such as idiopathic pulmonary fibrosis, ILD, systemic sclerosis, pneumocystis jivorecii pneumonia, community-acquired pneumonia, and viral respiratory infections [18,19,20,21,22,23]. KL-6 is associated with both lung fibrosis and inflammation, while SP-D is mainly associated with lung inflammation [24].

In ILD, due to its underlying pathology, these biomarkers enter the bloodstream when damage occurs to alveolar cells, leading to a steep rise in serum levels. Furthermore, it has been discovered that high levels of KL-6 and SP-D are a good indicator of how severe, advancing, and fatal ILD could be [25]. The increase in follow-up indicates a worsening patient’s health condition, and the mitigated levels of these markers could indicate improved health conditions in the patient [26,27,28,29]. Thus, they have been used as diagnostic markers to predict ILD severity and prognosis [19,30,31,32,33].

Additionally, most of the studies only examined these biomarkers separately at a single time point. In this prospective, longitudinal study, we assess the biomarker level’s prognostic capacity, identify the cut-off level for predicting death or a poor prognosis, and identify if there is an additive effect of KL-6 and SP-D in improving the prognostic power of the biomarkers. Additionally, we aim to study if changes in KL-6 and SP-D concentrations are associated with changes in pulmonary function. Next, we aim to understand whether the addition of spirometry and radiologic severity scores to these hematological biomarkers can help further improve the predictive capabilities for disease progression and mortality in ILD. 

## 2. Materials and Methods

### 2.1. Study Population

We conducted a longitudinal, observational study that was carried out on 44 patients with ILD visiting the Department of Respiratory Medicine and Department of Rheumatology, JSS Medical College & Hospital, a university-affiliated 1800-bed tertiary care hospital, from 1 November 2019 to 30 April 2021. This study was approved by the Institutional Ethics Committee of JSS Medical College, Mysuru (Approval number: JSS/MC/PG/5189/2019-20). Written informed consent was obtained from either the patient or their legal guardian.

A diagnosis of ILD was established by the pulmonologist. An experienced pulmonologist and radiologist reviewed all pulmonary imaging, including a chest X-ray and HRCT. Furthermore, the following data were collected: age, sex, medical history, clinical manifestations, arterial blood gas analysis (ABGs), chest X-ray findings, HRCT findings, PFT results, forced vital capacity (FVC), forced expiratory volume in 1 s (FEV1), FEV1/FVC ratio, and diffusing capacity of the lungs for carbon monoxide (DLCO). The subjects were invited back for a repeat examination and to document survival after 6 months of their initial visit. The blood was collected on two occasions: during the first contact with the study personnel and after 6 months of follow-ups among survivors. The blood was collected between 11 am to 1 pm for all the patients. Patients above 18 years of age diagnosed with ILD of known and unknown etiology were included in the study. Patients with other respiratory diseases and relevant missing data were excluded from the study.

### 2.2. Definitions of No Change, Improvement, and Disease Progression

No change in condition was defined as an FVC change of <10% and a DLCO change of <15%. Disease improvement was defined as an increase in FVC by ≥10% and/or an increase in DLCO by ≥15%. A decline in FVC by ≥10% and/or a decrease in DLCO by ≥15%, an acute exacerbation, or death during follow-up were considered signs of disease progression [15].

### 2.3. HRCT Evaluation

Within 24 h of drawing blood, HRCTs were carried out on the ILD patients. HRCT data acquisitions were obtained at 1.0–1.5 mm at 10-mm intervals at the end of inspiration from the lung apex to the base. Two independent radiologists, who were blind to the patient’s diagnoses and clinical prognoses, assessed the images. According to the Fleischner Society’s [34] definition of ground-glass attenuation (GGA), consolidation, traction bronchiectasis, or bronchiectasis and honeycombing, each radiologist independently evaluated the presence, extent, and distribution of CT findings. The two radiologists then reached a consensus on the findings.

### 2.4. Detection of Serum KL-6 and SPD

Five ml of blood was collected from patients by venipuncture and centrifuged at 3000 rpm for 10 min within 2 h after the collection of blood to isolate serum and store it at −80 degrees Celsius for further analysis. The serum KL-6 and surfactant protein D levels were measured by sandwich-type enzyme-linked immunosorbent assay (ELISA) kits according to the manufacturer’s instructions at the presentation and 6 months later. Measurement of KL6 and surfactant protein D (SPD) was done using commercially available ELISA kits (KL6: Catalogue no: SEA413Hu, Cloud-Clone Corp., Katy, TX, USA; and SPD: Catalogue no: SEB039Hu, Cloud-Clone Corp., Katy, TX, USA).

### 2.5. Statistical Analysis

The statistical analysis was performed employing Jamovi (v2.25, The Jamovi Project, SYD, AUS). After an initial descriptive analysis, a comparison of the differences between survivors and expired patients at baseline was performed. Categorical variables were presented as percentages. The normality of the data was assessed using the Shapiro–Wilk test. Continuous variables were presented as either mean ± standard deviation if they were normally distributed or median with their interquartile range if they were not normally distributed. Statistical significance was assessed by the chi-square test for categorical variables and by the Student’s *T* or Wilcoxon signed-rank test for continuous variables, depending on the distribution of the data. Similarly, Pearson’s r test for correlation was used for normally distributed data, while non-normally distributed data were assessed using Spearman’s rho test. To determine the association between lung function and the biomarkers KL6 and SPD in ILD patients, general linear regression adjusted for age, sex, and BMI was conducted. The models with low Akaike information criterion (AIC) values were included.

The Cox proportional hazards regression analyses were used to calculate the hazard ratio (HR), and the Kaplan-Meier method was used to draw up 180-day survival curves, while the survival rates were compared using the log-rank test. Furthermore, receiver operating characteristic (ROC) curve analysis was performed using the calculated values (determined by Youden’s index) for the area under the curve (AUC), sensitivity, specificity, odds ratio, and optimal cut-off values of KL-6 for the prediction of disease progression. A two-tailed *p*-value of < 0.05 was considered statistically significant.

## 3. Results

In our study, a total of 44 participants were included; among them, 34 patients were survivors, seven expired during the study, and three patients were lost to follow-up. The survivors were significantly younger when compared to the non-survivors [56.0 (49.0–69.0) vs. 76.0 (71.0–84.0); *p* < 0.01]. Details of the demographic and clinical characteristics of the participants are enumerated in Table 1.

### 3.1. Lung Function, Haematology, and Radiology

Non-survivors were reported to have significantly higher CT scores [22.0 (22.0–28.0) vs. 11.5 (8.0–16.0); *p* < 0.01], total leukocyte count (TLC) [12,100 (10,946.7–14,608.3) vs. 9350 (7460.0–11,322.5); *p* = 0.01], and absolute neutrophil count (ANC) [12,380.0 (8876.7–12,436.7) vs. 6465.0 (5348.3–8827.5); *p* < 0.01], while FEV1% [45.0 (37.0–45.0) vs. 70.0 (58.9–80.0); *p* < 0.01], FVC% [1.2 (1.2–1.6) vs. 2.0 (1.7–2.7); *p* < 0.01], DLCO [3.1 (2.9–3.1) vs. 4.4 (3.6–4.8); *p* < 0.01], partial pressure of arterial oxygen (PaO_2_) [48.0 (48.0–49.8) vs. 66.3 (57.9–70.2); *p* < 0.01], oxygen saturation (SO_2_) [77.0 (72.0–82.0) vs. 92.0 (85.9–95.0); *p* < 0.01] were significantly lower when compared to the survivors. Specifically, KL-6 [3990.4 (3490.0–4467.6) vs. 1083.4 (856.2–1668.5); *p* < 0.01] and SPD values [256.1 (217.9–260.0) vs. 178.7 (152.7–202.5); *p* < 0.01] were significantly higher in non-survivors as compared to survivors (Table 1). Among non survivors, we observed 3.5-fold and 1.5-fold higher serum concentrations in KL6 and SPD, respectively, as compared to survivors.

### 3.2. KL6, SP-D, and Progression of the Disease

The patients who expired during the study were reported to have the highest KL-6 levels (3990.4 (3490.0–4467.6)) at baseline and the time of inclusion in the study, followed by participants who progressed (1357.0 (822.6–1543.4)) in severity, followed by patients in status quo (stable) (1068 (842.2–1824.0)). SP-D levels in the expired patients were highest (256.1 (217.9–260.0)), followed by the patients who progressed in disease severity (191.2 (152.8–210.5)) and patients in status quo (stable) (178.6 (157.0–218.2)) (Table 2).

**Figure 1:**
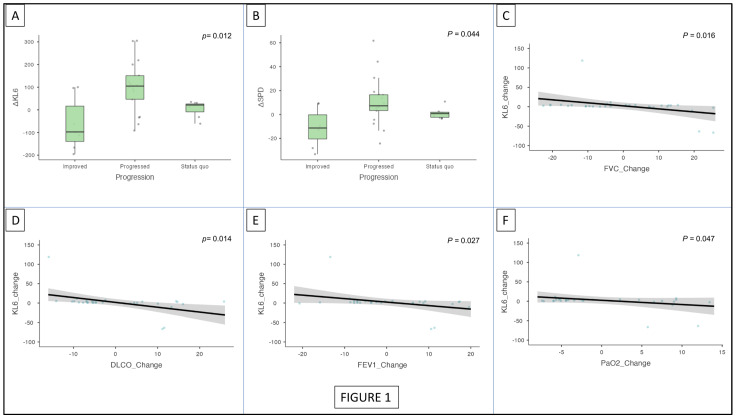
Trends in the ΔKL6 and ΔSPD in disease progression over 6 months of follow-up (**A**,**B**); generalized linear model analysis adjusted for age, sex, and BMI, showing an association between KL6 and FVC change, DLCO change, FEV1 change, and PaO_2_ change (**C**–**F**). KL-6: Krebs von den Lungen-6; SPD: surfactant protein D; FVC: forced vital capacity; DL_CO_: diffusing capacity of lungs for carbon monoxide; FEV1: forced expiratory volume in 1 s; PaO_2_: partial pressure of oxygen.

The differences in KL-6 and SP-D values pertinent to ΔKL-6 and ΔSPD following the 6-month follow-up were stratified based on disease progression as those who improved, worsened, or remained stable. In those patients who improved, ΔKL-6 and ΔSPD values were lowered by 97 (−140 to 16.5) and 11.3 (−20.5 to −0.2), respectively, while those patients who progressively worsened reported a rise in ΔKL-6 and ΔSPD values by 105 (46.6 to 151) and 9.38 (5.41 to 16.5), respectively. Patients who showed no changes in status quo (stable) showed minor variations, with an increase in KL-6 values by 21.5 (−8.97 to 16.5) and SP-D values by 0.8 (−2.3 to 1.95). Welch’s ANOVA test showed a statistically significant difference between the three groups for both ΔKL-6 (*p* = 0.012) and ΔSPD (*p* = 0.044) (Figure 1A,B).

**Figure 2:**
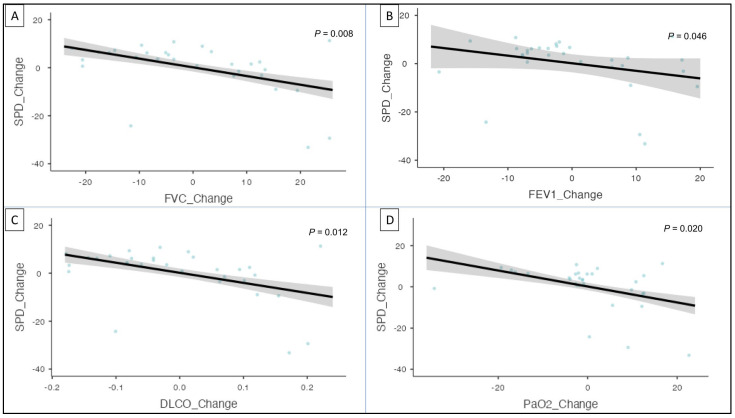
Generalized linear model analysis adjusted for age, sex, and BMI shows an association between SPD and FVC change, FEV1 change, DLCO change, and PaO_2_ change (**A**–**D**). SPD: surfactant protein D; FVC: forced vital capacity; FEV1: forced expiratory volume in 1 s; DL_CO_: diffusing capacity of lungs for carbon monoxide; PaO_2_: partial pressure of oxygen.

Furthermore, when adjusted for age, sex, and BMI, GLM analysis showed that ΔKL6 was associated with the change in FVC (*p* = 0.016), DLCO (*p* = 0.014), FEV1 (*p* = 0.027), and PaO_2_ (*p* = 0.047) (Figure 1C,F). Similarly, SPD was also associated with the change in FVC (*p* = 0.008), DLCO (*p* = 0.012), FEV1 (*p* = 0.046), and PaO_2_ (*p* = 0.020) on GLM analysis (Figure 2A–D).

### 3.3. Correlation Matrix between KL-6, SPD, Lung Functions, and Imaging Parameters 

Both KL-6 and SPD correlate negatively with DLCO (% pred) (KL-6: r = −0.587, *p* < 0.001; SPD: r = −0.417, *p* = 0.014), while both correlate positively with CT scores (KL-6: r = 0.710, *p* < 0.001; SPD: r = 0.609, *p* < 0.001). KL-6 and SPD correlated positively with each other (r = 0.547; *p* < 0.001). Time since diagnosis positively correlated with KL-6 (r = 0.425; *p* < 0.01), SPD (r = 0.430; *p* < 0.01), and CT scores (r = 0.488; *p* < 0.01) (Table 3). 

### 3.4. Prognostic Values of KL-6 and SPD

The ROC analysis was used to assess the prognostic value of KL-6, SP-D, FVC, SO_2_, and CT scores for predicting mortality and disease progression. 

**Figure 3:**
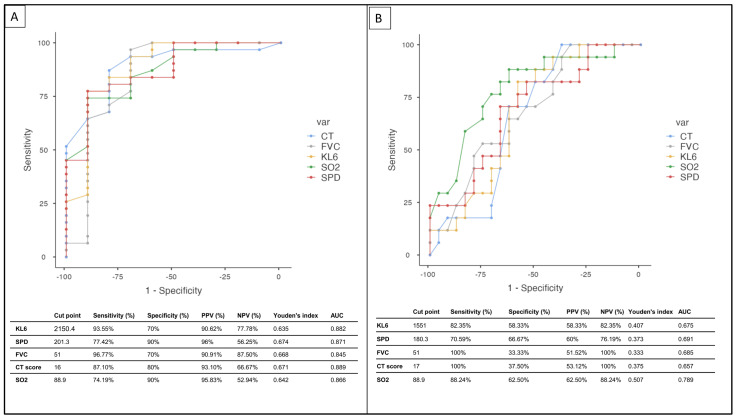
This figure depicts the results of ROC analysis for (**A**) KL-6, SP-D, FVC, CT scores, and SO_2_ for predicting mortality; (**B**) KL-6, SP-D, FVC, CT scores, and SO_2_ for disease progression. KL-6: Krebs von den Lungen-6; SPD: surfactant protein D; FVC: forced vital capacity; CT: computed tomography; SO_2_: oxygen saturation.

For mortality, the cut-off value for KL-6 was 2150.4 (SEN: 93.55%; SPE: 70%), and the cut-off points for SP-D, FVC, SO_2_, and CT score were found to be 201.3 (SEN: 77.42%; specificity: 90%), 51 (SEN: 96.77%; SPE: 70%), 88.9 (SEN: 74.19%; SPE: 90%), and 16 (SEN: 87.10%; SPE: 80%), respectively (Figure 3A). The power of the combined indicators (KL6 + SPD + CT + FVC) to predict disease mortality was the highest (SPE: 0.800; SEN: 0.935; AUC: 0.961) (Table 4).

For progression, the cut-off value for KL-6 was 1551 (SEN: 82.35%; SPE: 58.33%), and the cut-off points for SP-D, FVC, SO_2_, and CT scores were found to be 180.3 (SEN: 70.59%; SPE: 66.67%), 51 (SEN: 100%; SPE: 33.33%), 88.9 (SEN: 88.24%; SPE: 62.50%), and 17 (SEN: 100%; SPE: 37.50%), respectively (Figure 3B). The prognostic power of the combined indicators (KL6 + SPD + CT + FVC) to predict disease progression was the highest (SPE: 0.706; SEN: 0.750; AUC: 0.797) (Table 5).

### 3.5. Odds of Disease Progression

We performed a univariable logistic regression analysis to determine the odds of disease progression for each lung function parameter, age, and sex. According to our observation, higher CT severity score [OR(95%CI): 2.47 (1.55–3.93, *p* < 0.001)], KL-6 levels [OR(95%CI): 3.30 (1.25–8.72, *p* = 0.016)], and SPD levels [OR(95%CI): 2.21 (1.37–3.56, *p* = 0.001)] typically showed higher odds of disease progression, but higher FVC predicted [OR(95%CI): 0.56 (0.31–1.01, *p* = 0.056)], FEV1 predicted [OR(95%CI): 0.95 (0.90–0.99, *p* = 0.028)] and higher DLCO predicted [OR(95%CI): 0.70 (0.52–0.93, *p* = 0.013)] were reported to be protective against disease progression (Table 6). 

Additionally, to control for potential confounders, we performed multivariable logistic regression to estimate the odds of disease progression. We found that CT severity score [OR (95%CI): 3.89 (1.28–11.81, *p* = 0.017)], FVC predicted [OR (95%CI): 0.36 (0.16–0.82, *p* = 0.015)], DLCO predicted [OR (95%CI): 0.56 (0.33–0.97, *p* = 0.037)], KL-6 [OR (95%CI): 2.87 (1.06–7.79, *p* = 0.038)] and SPD [OR (95%CI): 1.76 (1.05–2.97, *p* = 0.033)] remained significant (Table 6). 

### 3.6. Risk of Mortality in ILD Patients

**Figure 4:**
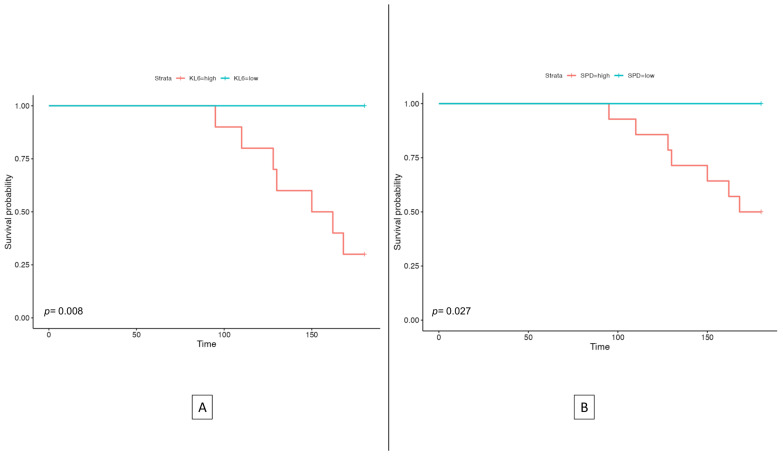
Kaplan-Meier survival curve to assess 180-day mortality of subjects with different KL6 levels (**A**) and subjects with different SPD levels (**B**).

A Kaplan–Meier analysis was performed to assess 180-day mortality in these patients with ILD segregated according to ROC cut-off values pertinent to KL-6 and SPD. Patients with a higher KL-6 (>2150.4) typically exhibited a significantly lower 180-day survival probability (*p* = 0.008) than patients with a lower KL-6 (<2150.4) (Figure 4A). Similarly, patients with higher SPD (>201.3) reported a significantly lower 180-day survival probability (*p* = 0.027) than patients with lower SPD (<201.3) (Figure 4B).

We performed a univariable Cox-regression analysis to assess the risk of mortality in our study population. Higher age [HR (95%CI): 1.16 (1.06–1.28, *p* = 0.002)], CT severity score [HR (95%CI): 1.64 (1.10–2.45, *p* = 0.015)], KL-6 [HR (95%CI): 2.18 (1.04–4.61, *p* = 0.040)] and SPD [HR (95%CI): 4.82 (2.29–10.16, *p* < 0.001)] levels predicted a higher hazard of mortality; while a higher FVC was predicted [HR (95%CI): 0.89 (0.84–0.94, *p* < 0.001)]. FEV1 predicted [HR (95%CI): 0.82 (0.73–0.91, *p* < 0.001)], DLCO predicted [HR (95%CI): 0.61 (0.27–0.84, *p* = 0.055)] PaO2 [HR (95%CI): 0.77 (0.65–0.91, *p* = 0.002)] and SO_2_ [HR (95%CI): 0.84 (0.76–0.93, *p* < 0.001)] levels predicted a low probability of mortality (Table 7).

However, we found that CT severity scores [HR (95%CI): 4.02 (1.04–15.60, *p* = 0.044)], FVC predicted [OR (95%CI): 0.88 (0.81–0.96, *p* = 0.005)], DLCO predicted [HR (95%CI): 10.60 (0.24–0.84, *p* = 0.047)], and KL-6 [HR (95%CI): 3.70 (1.46–9.41, *p* = 0.006)] and SPD [HR (95%CI): 2.58 (1.01–6.59, *p* = 0.047)] retained their significance in multivariable Cox-regression analysis after controlling for potential confounders (Table 7).

The majority of patients with idiopathic pulmonary fibrosis (57.2%) received nintedanib, while 42.8% received pirfenidone. All ILD patients with lung dominant connective tissue disorders received cyclophosphamide. In the case of rheumatoid arthritis, 50% of patients received azathioprine, and the other 50% received cyclophosphamide. All patients with systemic sclerosis received cyclophosphamide, and in the case of sarcoidosis, all patients received corticosteroids. For hypersensitivity pneumonitis, all patients received azathioprine. Overall, the treatments varied depending on the type of ILD, with cyclophosphamide being the most commonly used treatment in the cohort (Table S1).

## 4. Discussion

The findings of this study indicate that higher levels of KL-6 and SPD in the blood have a significant effect on the severity, disease progression, and mortality of ILD. This research is one of the few to follow prospectively, changes in KL-6 and SPD levels and their impact on ILD outcomes over time. An analysis that considered factors such as age, gender, and BMI found that changes in clinically relevant outcomes such as FVC, FEV1, DLCO, PaO_2_, and time since diagnosis were strongly related to changes in KL-6 and SPD levels. While KL-6 and SP-D are effective in predicting death, their ability to predict the progression of ILD is moderate when considered separately. However, the use of both biomarkers and lung function measurements together enhances the predictive power of ILD outcomes.

The results of our study found that those who succumbed to the disease had the highest serum levels of KL 6, with a mean difference of 2096 U/mL between those who survived and those who did not a three-fold difference. Other research has also reported similar high mean differences, averaging 1500 U/mL, and a two-to-three-fold difference between survivors and non-survivors [35,36]. Our study found that elevated KL-6 was a strong predictor of mortality, with a hazard ratio of 3.70 (1.46–9.41). This is consistent with other studies that have found hazard ratios ranging from 9.19 (1.60, 174.00) to 1.24 (1.05, 1.46) [37,38]. A meta-analysis of KL 6 as a biomarker for mortality in ILD patients, consisting of 43 studies, showed a pooled hazard ratio of 2.05 (1.50–2.78) [39]. Our study determined that the optimal cut-off value for KL 6 was 2150.4 U/mL, which is very similar to the value found by Satoh et al. (2750 U/mL). However, other studies have suggested lower cut-off values [27,40,41]. This discrepancy may be due to differences in the methods used to measure KL 6, as well as the diverse nature of ILD, which includes various diseases. Furthermore, the degree of lung damage caused by ILD, along with other factors such as ARDS and septic shock, can also affect the levels of KL 6 [42,43,44]. Although there are limited data on the optimal cut-off value for predicting mortality in ILD, a value of KL 6 greater than 1000 U/mL may be considered a reliable indicator to predict mortality [45].

In our study, the baseline KL-6 levels were higher in individuals with progressive ILD compared to those with stable ILD, with 2.87 times higher odds of disease progression. KL-6 levels showed a dynamic response to changes in the clinical status of the patients. After 6 months of treatment, KL-6 values increased by a 7.73% percentage in patients with progressive disease, decreased by a 10.94% percentage in patients who improved and increased marginally by 2.01% in stable patients. Only a few studies have repeated measurements of KL-6 levels during the course of the study. Zheng [46] found that in patients with progressive disease, KL-6 levels increased by 80% from 1070 IU/mL at baseline to 1875 IU/mL over 3 months, despite treatment, and KL-6 was negatively correlated with FVC and FEV1 changes over 3 months. In patients with stable disease, KL-6 values decreased from 1179 IU/mL to 1023 IU/mL after treatment. Yoshikawa [47] conducted a 6-month follow-up study and measured KL-6 levels at baseline, 3 months, and 6 months after treatment. They found that among patients with progressive disease, KL-6 increased by 30% at 3 months (901 IU/mL at baseline and 1195 IU/mL at 3 months) and 37% at 6 months (1237 IU/mL). In contrast, among patients with stable disease, there was a decrease in KL-6 levels of 12.5% (885 IU/mL at baseline to 775 IU/mL at 3 months) and a decrease of 16.5% at 6 months (738 IU/mL). They also found a negative correlation between the change in DLCO and the change in KL-6 over 6 months.

The results of our study show that increased levels of SPD have a significant impact on the mortality of ILD patients. We found that non-survivors had higher levels of SP-D (256.1 ng/mL) compared to survivors, with a significant difference in mean SP-D levels between the two groups. Takahashi et al. conducted two studies to examine the relationship between SP-D levels and survival outcomes in ILD patients. In the first study, they observed that non-survivors had significantly higher initial levels of SP-D (453.7 ± 290.3 ng/mL) compared to survivors (248.0 ± 176.4 ng/mL) [48]. In their second study, they found that patients with SP-D levels equal to or greater than 253 ng/mL had shorter survival than those with levels below this threshold during the five-year follow-up [49]. These findings support the importance of monitoring SP-D levels in the prediction of ILD outcomes. Our hazard ratio for SPD as a predictor of mortality was 2.58 (1.01–6.59), which is consistent with the results of other studies [50,51,52].

Our findings were consistent with a systematic review and meta-analysis conducted by Wang et al., which analyzed 21 studies and concluded that elevated levels of SPD increased the risk of mortality by 111% when compared to low SP-D levels and resulted in a higher HR of 2.11 (1.60–2.78) for poor prognosis [32]. The optimum cut-off value for SPD in our study was 201.3 ng/mL, which is similar to other studies [53]. However, the cut-off values for SPD can vary between studies and depend on the type of injury and its impact on lung permeability [27]. In some cases, the cut-off value may be higher, such as the 460 ng/mL observed by Barlo et al. [50] due to the direct leakage of SPD into the bloodstream through capillaries. These findings highlight the importance of measuring SPD levels in ILD patients as a predictor of mortality and as a potential treatment target.

In our study, we observed that progressive ILD exhibited the second-highest levels of SPD at 191.2 ng/mL, which was 12.6 ng/mL more than those of stable ILD and other groups in terms of disease progression. These higher SPD levels were associated with 1.76 times greater odds of predicting disease progression. Our study followed up with patients for 6 months, and even a small increase in SPD levels was associated with disease progression. The SP-D values of patients were monitored for six months, revealing an increase of 5.91% in patients whose disease progressed, a decrease of 7.16% in those who showed improvement, and an increase of 0.44% in stable patients. Previously, we could identify only one study that repeated SP-D levels during the course of treatment. Yoshikawa et al. [47] followed up with ILD patients for 6 months, and their SP-D levels were measured at baseline, 3 months into treatment, and 6 months. Results showed that for patients with progressive ILD, SP-D levels increased slightly by 6.1% at 3 months (261 ng/mL) before declining by 8.9% (to 224 ng/mL) compared to baseline (246 ng/mL). In contrast, patients with stable ILD experienced a significant reduction in SP-D levels, from 241 ng/mL at baseline to a 19.5% decrease (to 194 ng/mL) at 3 months and 19% (to 195 ng/mL) at the end of 6 months compared to baseline. They also observed a negative correlation between the changes in FVC, DLCO, and SP-D over the period of 6 months.

Our research revealed that both biomarkers were highly effective at predicting mortality, as confirmed by ROC analysis (AUC: SPD = 0.871; KL-6 = 0.882). However, when utilized as a prognostic indicator for disease progression, they were found to be inadequate. To address this issue, we employed a multi-indicator approach that combined KL-6, SPD, CT score, and FVC (% pred) (AUC: 0.797), resulting in significantly improved accuracy. While the practice of combining biomarkers to enhance prognostic accuracy has been utilized in other illnesses, such as community-acquired pneumonia [54,55], our study marks a pioneering investigation into this approach in ILD. It is a novel discovery that sets our study apart. Only one previous study examined a combined KL-6 and SP-D, concluding that it was a superior predictor than either biomarker alone [25]. This study did not combine CT scores and FVC for KL-6 and SP-D.

The use of KL-6 and SPD as biomarkers for assessing the current status and monitoring the progression of ILD has been found to be very useful in our study. These biomarkers can be easily measured via a simple blood test, providing a convenient and non-invasive alternative to chest HRCT for ILD [56], which has high exposure to radiation and cannot be performed frequently. Studies have demonstrated that serial measurements of KL-6 and SPD are useful in predicting disease progression, exacerbation of ILD, and mortality. Combining regular chest HRCT with more frequent measurement of these biomarkers could potentially serve as an ideal protocol for clinical monitoring for disease progression, risk of exacerbation, and death among ILD patients and can be cost-effective and safer compared to frequent HRCT of the thorax. However, further research is necessary to determine the optimal timing and frequency of biomarker measurements in clinical practice.

Our study has certain limitations. Firstly, the sample size was limited, and the scope of ILD encompasses a broad spectrum of causative factors. Therefore, the study results may not be generalizable and need a larger sample and subgroup analysis to confirm our findings. Secondly, most of the patients had either moderate or severe forms of the disease, and there was insufficient data on those with mild ILD, potentially limiting the accuracy of the findings regarding the biomarkers and their relationship with prognosis in patients with mild ILD. Thirdly, due to the diverse etiologies and severity levels, the treatment received by the participants was not uniform. Therefore, future research should consider these factors and investigate their implications with appropriate stratification. In subsequent studies, a more extended follow-up period would be beneficial.

## 5. Conclusions

We observed that higher KL-6 and SPD levels are important biomarkers that might predict the severity, progression, and outcomes of ILD. Clinicians should be aware of the possibility of patients having a poor prognosis when KL-6 and SPD baseline levels are high or increase over the six-month follow-up period, despite their treatment, and there is a need for clinical studies to study additional interventions that could benefit the patients when there is a possibility of treatment failure. We developed a more potent prognostic indicator to measure disease progression by combining KL6 and SPD with more conventional measures such as CT scores and FVC. Future research is required to determine whether this combined biomarker is beneficial to different ethnicities across the globe.

## Figures and Tables

**Table 1:** Baseline characteristics of study samples stratified based on survival.

	Survivor (n = 34)	Expired (n = 7)	*p*-Value
Age in years	56.0 (49.0–69.0)	76.0 (71.0–84.0)	<0.01 *
Male (n, %)	18 (52.94)	5 (71.4)	
Female (n, %)	16 (47.06)	2 (28.6)	0.37 ^#^
Time Dx	12.0 (1.8–24.0)	22.0 (22.0–22.8)	0.04 *
Vitals, Pulmonary Physiology, Radiology			
CT Severity	11.5 (8.0–16.0)	22.0 (22.0–22.8)	<0.01 *
FVC	2.0 (1.7–2.7)	1.2 (1.2–1.6)	<0.01 *
FVC predicted %	70.0 (62.0–78.0)	42.0 (42.0–42.0)	<0.01 *
FEV1	1.6 (1.4–2.1)	1.0 (0.8–1.0)	<0.01 *
FEV1 predicted %	70.0 (58.9–80.0)	45.0 (37.0–45.0)	<0.01 *
FEV1/FVC ratio	0.8 (0.8–0.8)	0.8 (0.6–0.8)	0.15 *
DL_CO_	4.4 (3.6–4.8)	3.1 (2.9–3.1)	<0.01 *
DL_CO_ predicted %	60.5 (48.0–66.1)	40.0 (37.2–40.0)	<0.01 *
EF	60.0 (60.0–60.0)	60.0 (58.3–61.7)	0.85 *
PH	7.4 (7.4–7.4)	7.4 (7.4–7.4)	0.28 *
PaO_2_ (mmHg)	66.3 (57.9–70.2)	48.0 (48.0–49.8)	<0.01 *
PCO_2_ (mmHg)	34.0 (32.9–37.0)	40.0 (32.7–41.7)	0.24 *
HCO_3_ (mmol/l)	23.5 (22.0–24.0)	25.0 (22.0–25.7)	0.42 *
SO_2_ (%)	92.0 (85.9–95.0)	77.0 (72.0–82.8)	<0.01 *
Hematological investigations			
Hemoglobin (g/dL)	13.0 (11.7–14.2)	14.2 (11.2–14.9)	0.56 *
KL-6 (U/mL)	1083.4 (856.2–1668.5)	3990.4 (3490.0–4467.6)	<0.01 *
SPD (ng/mL)	178.7 (152.7–202.5)	256.1 (217.9–260.0)	<0.01 *
TC	9350.0 (7460.0–11,322.5)	12,100.0 (10,946.7–14,608.3)	0.01 *
Absolute Neutrophil count	6465.0 (5348.3–8827.5)	12,380.0 (8876.7–12,436.7)	<0.01 *
Absolute Lymphocyte count	1550.0 (1099.2–2263.3)	2610.0 (1521.7–2618.3)	0.30 *
Platelet	265,000.0 (212,750.0–330,000.0)	350,000.0 (227,500.0–500,166.7)	0.24 *
NLR	4.3 (2.8–7.7)	4.7 (3.4–8.2)	0.39 *
PLR	165.4 (122.5–266.8)	132.5 (86.9–356.6)	0.71 *
RDW	13.0 (12.0–15.0)	13.6 (11.4–14.8)	0.82 *

^#^ Pearson. * Wilcoxon. Time Dx: Time since diagnosis; CT: computed tomography; FVC: forced vital capacity; FEV1: forced expiratory volume in 1 s; DL_CO_: diffusing capacity of lungs for carbon monoxide; EF: ejection fraction; PaO_2_: partial pressure of oxygen; PCO_2_: partial pressure of carbon dioxide; HCO_3_: bicarbonate; SO_2_: oxygen saturation; KL-6: Krebs von den Lungen-6; SPD: surfactant protein D; PH: acidity/alkalinity; TC: total leukocyte count; NLR: neutrophil-lymphocyte ratio; PLR: platelet-lymphocyte ratio; RDW: red cell distribution width.

**Table 2:** Baseline characteristics of study samples stratified based on progression.

	Status Quo (n = 7)	Progressed (n = 17)	Improved (n = 7)	Expired (n = 7)	*p*-Value
Age in years	61.0 (58.3–67.8)	56.0 (44.7–69.3)	51.0 (44.0–62.7)	76.0 (71.0–84.0)	0.13 ^3^
Male (n, %)	4 (57.1)	8 (47.1)	5 (71.4)	5 (71.4)	0.64 ^2^
Female (n, %)	3 (42.9)	9 (52.9)	2 (28.6)	2 (28.6)	
BMI	22.6 (22.0–23.0)	21.0 (18.1–23.0)	21.0 (21.0–22.8)	22.0 (21.0–22.8)	0.65 ^3^
Time Dx	24 (16.5–24.0)	12.0 (5.3–24.0)	8.0 (0.3–17.3)	22.0 (22.0–22.8)	0.06 ^3^
Vitals					
CT Severity	9.0 (2.7–11.7)	13.0 (8.0–18.7)	12.0 (10.0–15.7)	22.0 (22.0–22.8)	<0.01 ^3^
FVC	1.8 (1.7–2.1)	2.1 (1.7–2.6)	2.7 (1.8–3.0)	1.2 (1.2–1.6)	0.10 ^3^
FVC predicted %	74.0 (65.5–79.5)	70.0 (62.0–77.3)	75.0 (62.8–79.7)	42.0 (42.0–42.0)	<0.01 ^3^
FEV1	1.4 (1.4–1.8)	1.7 (1.4–2.0)	2.1 (1.4–2.3)	1.0 (0.8–1.0)	0.02 ^3^
FEV1 predicted %	69.0 (63.0–85.3)	62.0 (58.0–78.7)	77.0 (71.0–77.8)	45.0 (37.0–45.0)	<0.01 ^3^
DL_CO_	4.4 (3.6–4.8)	4.3 (3.6–4.8)	4.4 (3.9–4.6)	3.1 (2.9–3.1)	0.02 ^3^
DL_CO_ predicted %	55.0 (42.3–68.0)	62.0 (47.7–66.3)	59.0 (55.5–65.8)	40.0 (37.2–40.0)	0.03 ^3^
EF	60.0 (60.0–62.0)	60.0 (58.0–60.0)	60.0 (60.0–60.0)	60.0 (58.3–61.7)	0.38 ^3^
PH	7.4 (7.4–7.4)	7.4 (7.4–7.4)	7.4 (7.4–7.4)	7.4 (7.4–7.4)	0.12 ^3^
PaO_2_ (mmHg)	67.0 (62.0–69.8)	58.0 (55.0–66.5)	72.0 (58.8–89.0)	48.0 (48.0–49.8)	0.06 ^3^
PCO_2_ (mmHg)	34.0 (33.7–35.9)	35.0 (32.7–39.1)	35.0 (31.2–37.8)	40.0 (32.7–41.7)	0.48 ^3^
HCO_3_ (mmol/l)	23.0 (21.3–24.0)	23.0 (21.8–24.1)	24.0 (23.0–24.8)	25.0 (22.0–25.7)	0.20 ^3^
SO_2_ (%)	94.0 (90.3–95.7)	88.0 (83.7–93.3)	94.0 (86.3–95.8)	77.0 (72.0–82.8)	0.02 ^3^
Hematological investigations					
Hemoglobin (g/dL)	13.2 (12.3–14.2	12.3 11.5–14.3)	13.0 (11.8–14.0	14.2 (11.2–14.9)	0.95 ^3^
KL-6 (U/mL)	1068.8 (842.2–1824.0)	1357.0 (822.6–1543.4)	904.4 (812.5–1203.7)	3990.4 (3490.0–4467.6)	<0.01 ^3^
SPD (ng/mL)	178.6 (157.0–218.2)	191.2 (152.8–210.5)	157.7 (135.2–180.1)	256.1 (217.9–260.0)	<0.01 ^3^
TC	8760.0 (6668.3–9565.0)	9250.0 7633.3–12,570.0)	9860.0 (9503.3–10,638.3)	12,100.0 (10,946.7–14,608.3)	0.02 ^3^
ANC	5630.0 (4836.7–6466.7)	6960.0 (5360.0–9426.7)	7740.0 (6223.3–8670.0)	12,380.0 (8876.7–12,436.7)	0.01 ^3^
ALC	1980.0 (1720.0–2283.3)	1240.0 (1033.3–1803.3)	2260.0 (1146.7–2690.0)	2610.0 (1521.7–2618.3)	0.70 ^3^
Platelet	308,000.0 (235,000.0–318,333.3)	270,000.0 (212,000.0–323,333.3)	230,000.0 (219,166.7–388,333.3)	350,000.0 (227,500.0–500,166.7)	0.51 ^3^
NLR	2.9 (2.0–4.1)	4.3 (3.7–11.3)	4.4 (2.1–7.0)	4.7 (3.4–8.2)	0.18 ^3^
PLR	153.2 (102.0–156.2)	209.6 (135.0–335.7)	125.0 (77.9–352.2)	132.5 (86.9–356.6)	0.89 ^3^
RDW	13.0 (11.5–16.3)	13.3 (12.5–15.3)	13.0 (12.1–14.5)	13.6 (11.4–14.8)	0.89 ^3^

^2^ Pearson. ^3^ Wilcoxon. BMI: Body mass index; Time Dx: Time since diagnosis; CT: computed tomography; FVC: forced vital capacity; FEV1: forced expiratory volume in 1 s; DL_CO_: diffusing capacity of lungs for carbon monoxide; EF: Ejection fraction; PaO_2_: partial pressure of oxygen; PCO_2_: partial pressure of carbon dioxide; HCO_3_: Bicarbonate; SO_2_: oxygen saturation; KL-6: Krebs von den Lungen-6; SPD: surfactant protein D; PH: acidity/alkalinity; TC: total leukocyte count; ANC: Absolute Neutrophil count; ALC: Absolute Lymphocyte count; NLR: neutrophil-lymphocytes ratio; PLR: platelet-lymphocyte ratio; RDW: red cell distribution width.

**Table 3:** Correlation Matrix Between KL-6, SPD, Lung Functions, And Imaging Parameters.

	KL-6	SPD	CT Severity	FVC Predicted %	FEV1 Predicted %	DL_CO_ Predicted %	Time_Dx
KL-6	—						
SPD	0.547 ***	—					
CT Severity	0.710 ***	0.609 ***	—				
FVC predicted %	−0.689 ***	−0.473 **	−0.491 ***	—			
FEV1 predicted %	−0.583 ***	−0.411 **	−0.355 *	0.804 ***	—		
DL_CO_ predicted %	−0.587 ***	−0.417 **	−0.471 **	0.701 ***	0.649 ***	—	
Time_Dx	0.425 **	0.430 **	0.488 **	−0.153	−0.247	−0.090	—

KL-6: Krebs von den Lungen-6; SPD: surfactant protein; CT: computed tomography; FVC: forced vital capacity; FEV1: forced expiratory volume in 1 s; DLCO: diffusing capacity of lungs for carbon monoxide; Time Dx: time since diagnosis. * *p* < 0.05, ** *p* < 0.01, *** *p* < 0.001.

**Table 4:** Predictive Measures for a combination of biomarkers and lung parameters for disease mortality.

Indicator	Accuracy	Specificity	Sensitivity	AUC
KL6 only	0.854	0.700	0.935	0.882
SP-D only	0.805	0.900	0.774	0.871
KL6 + SPD	0.854	0.500	0.968	0.884
KL6 + SPD + CT	0.878	0.800	0.903	0.942
KL6 + SPD + CT + FVC	0.902	0.800	0.935	0.961

The cut-off value is set to 0.5, KL-6: Krebs von den Lungen-6; SPD: surfactant protein; CT: computed tomography; FVC: forced vital capacity.

**Table 5:** Predictive Measures for a combination of biomarkers and lung parameters for disease progression.

Indicator	Accuracy	Specificity	Sensitivity	AUC
KL6 only	0.585	0.583	0.823	0.675
SP-D only	0.592	0.667	0.705	0.691
KL6 + SPD	0.610	0.529	0.667	0.701
KL6 + SPD + CT	0.634	0.588	0.667	0.716
KL6 + SPD + CT + FVC	0.732	0.706	0.750	0.797

The cut-off value is set to 0.5; KL-6: Krebs von den Lungen-6; SPD: surfactant protein; CT: computed tomography; FVC: forced vital capacity.

**Table 6:** Univariable and Multivariable logistic regression analysis to determine the odds of disease progression.

Dependent: Progression	OR (Univariable)	OR (Multivariable)
Age	1.02 (0.97–1.07)	0.96 (0.88–1.04)
BMI	0.78 (0.55–1.05)	0.70 (0.44–1.00)
Female (n, %)	Reference	Reference
Male (n, %)	1.21 (0.34–4.35)	1.15 (0.31–4.38)
Time_Dx	1.14 (1.03–1.34) *	1.18 (0.79–1.98)
CT Severity	2.47 (1.55–3.93) ***	3.89 (1.28–11.81) *
FVC predicted %	0.56 (0.31–1.01)	0.36 (0.16–0.82) *
FEV1 predicted %	0.95 (0.90–0.99) *	0.96 (0.88–1.04)
DL_CO_ predicted %	0.70 (0.52–0.93) *	0.56 (0.33–0.97) *
KL-6 (U/mL)	3.30 (1.25–8.72) *	2.87 (1.06–7.79) *
SPD (ng/mL)	2.21 (1.37–3.56) **	1.76 (1.05–2.97) *
TC	1.00 (1.00–1.00) *	1.00 (1.00–1.00)
ANC	1.00 (1.00–1.00) *	1.00 (1.00–1.00)

* *p* = < 0.05, ** *p* = < 0.01, *** *p* = < 0.001, OR: odds ratio, Time Dx: Time since diagnosis; CT: computed tomography; FVC: forced vital capacity; FEV1: forced expiratory volume in 1 s; DLCO: diffusing capacity of lungs for carbon monoxide; KL-6: Krebs von den Lungen-6; SPD: surfactant protein D; TC: total leukocyte count; ANC: absolute neutrophil count.

**Table 7:** Cox regression analysis of risk factors associated with ILD-related mortality.

Dependent: Death	HR (Univariable)	HR (Multivariable)
Female	Reference	Reference
Male	0.49 (0.10–2.55)	0.33 (0.04–2.49)
Age	1.16 (1.06–1.28) **	1.03 (0.84–1.25)
BMI	1.06 (0.75–1.50)	1.41 (0.71–2.80)
CT Severity	1.64 (1.10–2.45) *	4.02 (1.04–15.60) *
FVC predicted	0.89 (0.84–0.94) ***	0.88 (0.81–0.96) **
FEV1 predicted	0.82 (0.73–0.91) ***	0.99 (0.81–1.22)
DLCO	0.61 (0.27–0.84)	0.60 (0.24–0.84) *
PaO_2_	0.77 (0.65–0.91) **	1.02 (0.76–1.37)
SO_2_	0.84 (0.76–0.93) ***	0.85 (0.59–1.21)
KL6	2.18 (1.04–4.61) *	3.70 (1.46–9.41) **
SPD	4.82 (2.29–10.16) ***	2.58 (1.01–6.59) *
TC	1.09 (0.72–1.66)	1.16 (0.74–1.82)
ANC	1.05 (0.79–1.41)	1.07 (0.82–1.40)

* *p* = < 0.05, ** *p* = < 0.01, *** *p* = <0.001, HR: Hazards Ratio; CT: computed tomography; FVC: forced vital capacity; FEV1: forced expiratory volume in 1 s; DLCO: diffusing capacity of lungs for carbon monoxide; PaO_2_: partial pressure of oxygen; SO_2_: oxygen saturation; KL-6: Krebs von den Lungen-6; SPD: surfactant protein D; TC: total leukocyte count; ANC: absolute neutrophil count.

## Data Availability

All data generated or analyzed during this study are included in this published article and are available from the corresponding author upon reasonable request.

## Supplementary Materials

The following supporting information can be downloaded at: https://www.mdpi.com/article/10.3390/cells12091281/s1, Table S1: Treatments received by patients in the cohort at any time during the course of the study period.
