# Supplementary material for: Circulatory Serum Krebs von Den Lungen-6 and Surfactant Protein-D Concentrations Predict Interstitial Lung Disease Progression and Mortality
Source: Cells. 2023 Apr 28;12(9):1281. doi: 10.3390/cells12091281 (PMC10177381; doi:10.3390/cells12091281)
Supplement: Supplementary file 1 [file cells-12-01281-s001.zip › cells-2268664-supplementary.pdf]

Article

Circulatory Serum Krebs Von Den Lungen-6 and Surfactant Protein-D concentrations Predict Interstitial Lung Disease Progression and Mortality: Supplementary material

Meghna Rai[1], Ashwaghosha Parthasarathi[2, 3], Narasimha M Beeraka[4,5], Mohammed Kaleem Ullah[6,7], Sowmya Mala-mardi [1,8], Sunag Padukudru[9], Jayaraj Biligere Siddaiah[1], Chinnappa A. Uthaiah[6], Prashant Vishwanath[6], Sind-aghatta Krishnarao Chaya[1], Subramanian Ramaswamy[10], Swapna Upadhyay[11], Koustav Ganguly[11*] and Padukudru Anand Mahesh[1*]

1. Department of Respiratory Medicine, JSS Medical College, JSS Academy of Higher Education and Research, Mysuru 570015, India.
2. Allergy, Asthma, and Chest Centre, Krishnamurthypuram, Mysuru 570004, India.
3. Rutgers Centre for Pharmacoepidemiology and Treatment Science, New Brunswick, NJ 08901-1293, USA.
4. Department of Pharmaceutical Chemistry, JSS College of Pharmacy, JSS Academy of Higher Education and Research, Mysuru 570015, India.
5. Raghavendra Institute of Pharmaceutical Education and Research (RIPER), Anantapuramu, Chiyyedu, Andhra Pradesh 515721, India
6. Centre for Excellence in Molecular Biology and Regenerative Medicine (A DST-FIST Supported Center), Department of Biochemistry (A DST-FIST Supported Department), JSS Medical College, JSS Academy of Higher Education and Research, Mysore 570015, India.
7. Division of Infectious Disease and Vaccinology, School of Public Health, University of California, Berkeley, CA 94720, USA
8. School of Psychology & Public Health, College of Science Health and Engineering, La Trobe University, Melbourne, 3086, Australia.
9. Yenepoya Medical College, Yenepoya University, Mangalore, Karnataka, India.
10. Department of Clinical Immunology & Rheumatology, JSS Medical College, JSS Academy of Higher Education and Research, Mysuru 570015, India.
11. Unit of Integrative Toxicology, Institute of Environmental Medicine (IMM), Karolinska Institutet, 17177 Stockholm, Sweden.

* equal contribution and corresponding authors.

* Correspondence: PAM: pamahesh@jssuni.edu.in; K.G.: koustav.ganguly@ki.se

Table S1: Treatments received by patients in the cohort at any time during the course of the study period

Type of ILD	Treatment received	Patients (N=41)
Idiopathic Pulmonary Fibrosis	Nintedanib	8 (57.2%)
	Pirfenidone	6 (42.8%)
Lung dominant connective tissue disorder	Cyclophosphamide	10 (100%)
Rheumatoid Arthritis	Azathioprine	4 (50%)
	Cyclophosphamide	4 (50%)
Systemic Sclerosis	Cyclophosphamide	5 (100%)
Sarcoidosis	Corticosteroids	2 (100%)
Hypersensitivity pneumonitis	Azathioprine	2 (100%)
